# Common bean (*Phaseolus vulgaris* L.) α‐amylase inhibitors as safe nutraceutical strategy against diabetes and obesity: An update review

**DOI:** 10.1002/ptr.7480

**Published:** 2022-04-29

**Authors:** Stefania Peddio, Alessandra Padiglia, Faustina B. Cannea, Roberto Crnjar, Wissam Zam, Javad Sharifi‐Rad, Antonio Rescigno, Paolo Zucca

**Affiliations:** ^1^ Department of Biomedical Sciences (DiSB) Cittadella Universitaria di Monserrato Cagliari Italy; ^2^ Department of Life and Environmental Sciences (DiSVA) Cittadella Universitaria di Monserrato Cagliari Italy; ^3^ Department of Analytical and Food Chemistry, Faculty of Pharmacy Al‐Wadi International University Tartous Syria; ^4^ Facultad de Medicina Universidad del Azuay Cuenca Ecuador

**Keywords:** amylase inhibitor, bean, diabetes, obesity, *Phaseolus vulgaris*

## Abstract

Overweight and obesity are constantly increasing, not only in Western countries but also in low‐middle‐income ones. The decrease of both the intake of carbohydrates and their assimilation are among the main dietary strategies to counter these conditions. α‐Amylase, a key enzyme involved in the digestion of carbohydrates, is the target enzyme to reduce the absorption rate of carbohydrates. α‐Amylase inhibitors (α‐AIs) can be found in plants. The common bean, *Phaseolus vulgaris* is of particular interest due to the presence of protein‐based α‐AIs which, through a protein–protein interaction, reduce the activity of this enzyme. Here we describe the nature of the various types of common bean seed extracts, the type of protein inhibitors they contain, reviewing the recent Literature about their molecular structure and mechanism of action. We also explore the existing evidence (clinical trials conducted on both animals and humans) supporting the potential benefits of this protein inhibitors from *P. vulgaris*, also highlighting the urgent need of further studies to confirm the clinical efficacy of the commercial products. This work could contribute to summarize the knowledge and application of *P. vulgaris* extract as a nutraceutical strategy for controlling unwanted weight gains, also highlighting the current limitations.

**Abbreviations**
BMI: body mass index
GI: glycemic index
PHA: phytohemoagglutinins
PPA: porcine pancreatic α‐amylase
WL: weight loss
WM: weight maintenance
α‐AI: α‐amylase inhibitor

## 1 INTRODUCTION

Overweight and obesity are one of the major health concerns of our times, being considered the fifth leading risk for global deaths worldwide (The European Association for the Study of Obesity, 2021). According to WHO, more than 1.9 billion adults were overweight in 2016, and over 650 million of these were obese. Even among children and adolescents, obesity is a serious problem; over 340 million children and adolescents aged 5–19 were overweight or obese in 2016 with an increase of 14% from 1975 (WHO, 2021). It is estimated that globally there are more people obese than underweight in every region, except for some parts of sub‐Saharan Africa and Asia.

The imbalance between energy expenditure and diet calorie intake has been identified as the cause of this condition, being mainly caused by the decrease in physical activity and the shift toward diets rich in sugars and fats, and low in fibers and micronutrients (vitamins, minerals and so on) (Barrett & Udani, 2011).

Besides, overweight and obesity are related to many common health consequences, increasing risk for cardiovascular diseases, hypertension, type 2 diabetes (Meigs et al., 2006), dyslipidemia, sleep apnea, knee osteoarthritis (Raud et al., 2020), and certain types of cancer (Taroeno‐Hariadi, Hardianti, Sinorita, & Aryandono, 2021). More recently, obesity has even been identified as a leading risk factor for hospitalization and poor clinical outcome of SARS‐CoV2 patients during COVID‐19 pandemic (Mohammad et al., 2021).

Several dietary strategies have been suggested to tackle these issues, affecting carbohydrate and lipid metabolism. The most straightforward solution could be to reduce the carbohydrate portions or their absorption introducing dietary fibers (Bell & Sears, 2003). However, most people distaste such dietary modifications and report gastrointestinal issues (Udani, Tan, & Molina, 2018). Similar results can be obtained by inserting in the diet starches that resist digestion in the small intestine, thus acting like dietary fibers (Barrett & Udani, 2011). These starches are naturally found in several seeds, legumes and unprocessed whole grains, with a low glycemic index (GI).

A different approach could involve the use of some phytochemicals to slow down carbohydrate absorption. This could be achieved by preventing their intestinal hydrolysis, inhibiting the necessary enzymatic activity, namely amylase and glucosidase (Fukagawa, Anderson, Hageman, Young, & Minaker, 1990; Kaur et al., 2021).

**FIGURE 1:**
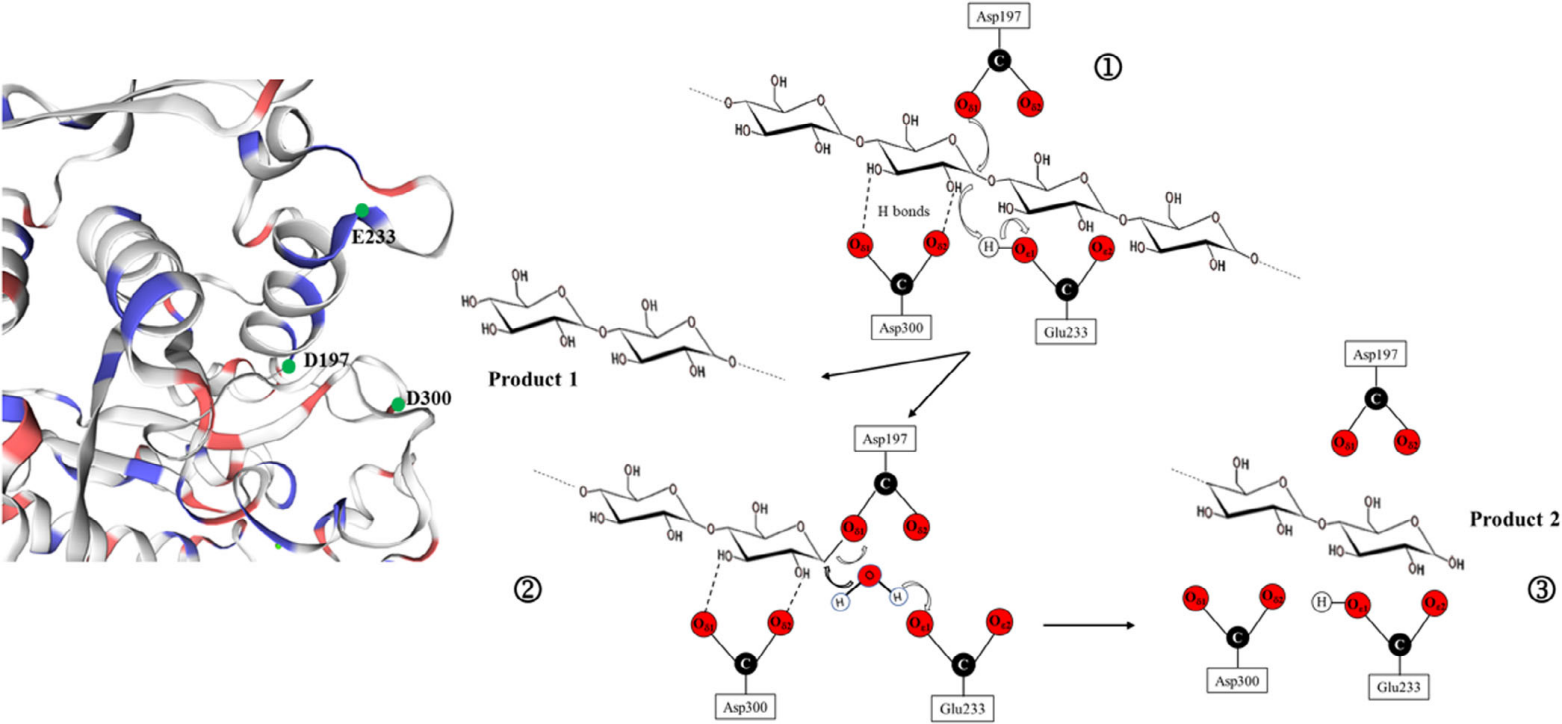
Reaction mechanism of PPA. (1) Nucleophilic attack of carboxylate group of Asp197 onto C1 of the α‐(1–4) glycosidic linkage and release of the first product; (2) hydrolysis of the β‐carboxylacetal covalent intermediate; (3) Release of the second product in α‐anomeric configuration. PPA, porcine pancreatic α‐amylase

α‐Amylase (α‐1,4‐glucan‐4‐glucanohydrolases, E.C. 3.2.1.1) is a glycoprotein catalyzing the endohydrolysis of (1→4)‐α‐d‐glycosidic linkages in polysaccharides containing three or more (1→4)‐α‐linked d‐glucose units (Kandra, 2003). During the reactions catalyzed by mammalian and bacteria α‐amylases, as shown in Figure 1, the formation of covalent β‐linked carbohydrate–enzyme intermediates in which are involved two aspartate residues and one residue of glutamate occurs (Hasegawa, Kubota, & Matsuura, 1999; Yoon, Bruce Fulton, & Robyt, 2007).

The human isoform is a single peptide chain of 512 amino acids (about 57 kDa), requiring calcium ions for structural integrity (Saboury & Karbassi, 2000; Whitcomb & Lowe, 2007). This is one of the major secretory enzymes of the salivary glands and pancreas, playing a central role in the digestion of starch and the absorption of deriving simple sugars. The cleavage of α‐d‐(1–4) glycosidic bonds releases shorter oligosaccharides retaining the α‐anomeric configuration, called dextrins (a mixture of maltose, maltotriose, and branched oligosaccharides of 6–8 glucose units with both α‐1,4 and α‐1,6 linkages). Other intestinal brush border enzymes (namely α‐glucosidase, maltase, and isomaltase) in turn further hydrolyze dextrins, and the resulting sugars can then be absorbed. No terminal glucose or α‐d‐(1–6) glycosidic bonds are affected by amylase activity (de Sales, de Souza, Simeoni, de Magalhães, & Silveira, 2012). Structurally, all α‐amylases are usually folded into three domains: A, B, and C. Domain A consists of a (β/α)_8_‐barrel, which is the core structure of the enzyme. Domain B is a sheet of four anti‐parallel β‐strands with a pair of anti‐parallel β‐strands. The binding site for calcium ion is located in this domain. Domain C consists of eight β‐strands forming a Greek key motif.

Thus, α‐amylase represents the crucial step in the initiation of the process of digestion and absorption of dietary polysaccharides (Tangphatsornruang, Naconsie, Thammarongtham, & Narangajavana, 2005). Therefore it can be the target for specific enzyme inhibitors, allowing to reduce the rate of carbohydrate absorption, the real energetic intake after a meal, and the subsequent glycemic peak (de Sales et al., 2012; Li et al., 2021).

**FIGURE 2:**
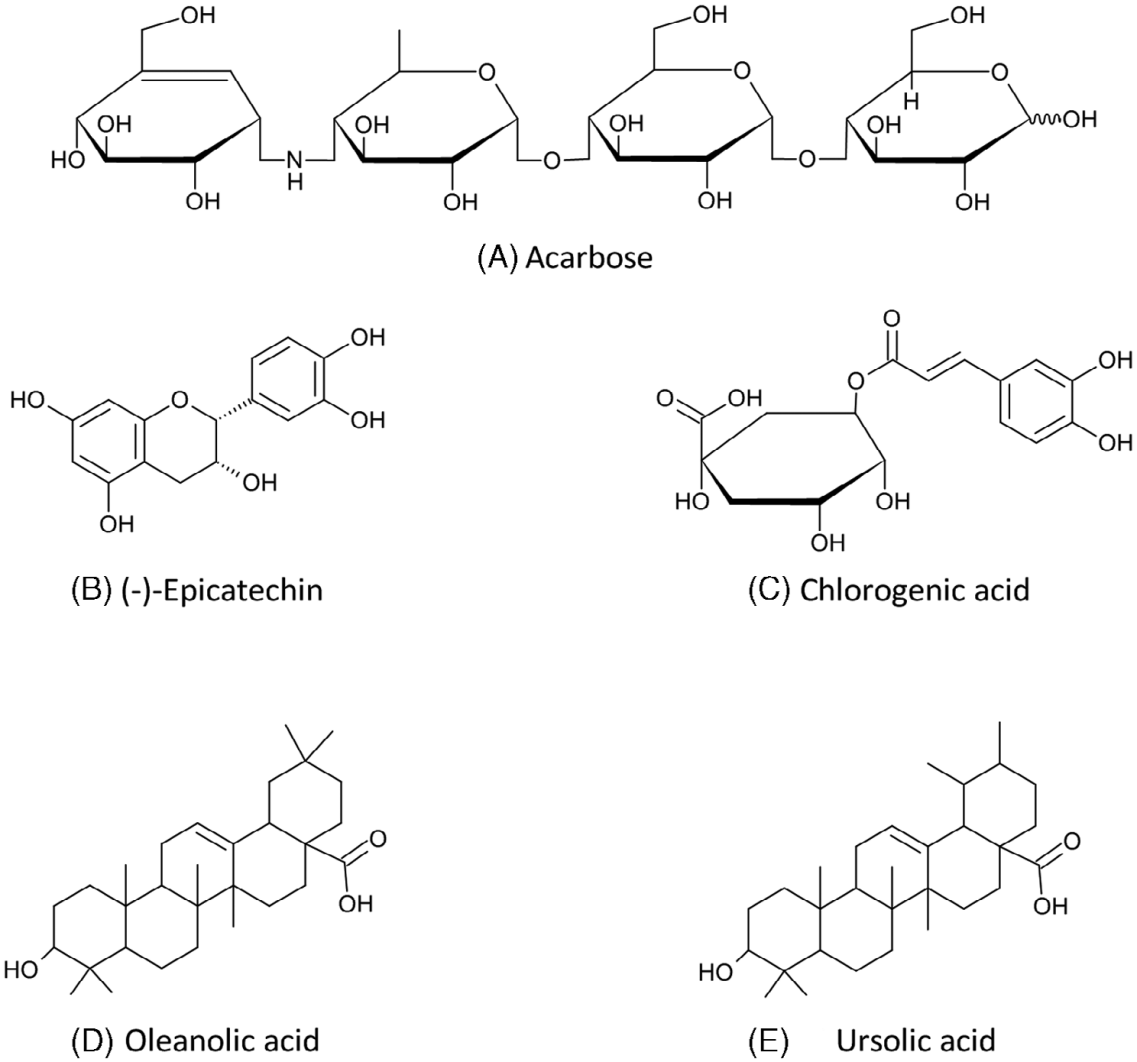
Some of the described low‐MW α‐amylase inhibitors

Several alternatives have been proposed: acarbose (commercially known as Prandase®, Glucobay®, Precose®) is the best known α‐amylase inhibitor (α‐AI). This is a low‐MW synthetic tetrasaccharide analog (Figure 2) used as a prescription drug to tackle hyperglycemia in the treatment of type 2 diabetes mellitus (Barrett & Udani, 2011). Miglitol and voglibose are other drugs belonging to the same class. As shown by several clinical studies, acarbose effectively improves insulin sensitivity in subjects with impaired glucose tolerance or type‐2 diabetes, reducing cardiovascular risks in subjects with metabolic syndrome (a cluster of risk factors including high triglycerides, low high‐density lipoprotein cholesterol, and hypertension) (Barrett & Udani, 2011; Yamagishi, Matsui, Ueda, Fukami, & Okuda, 2009).

Several α‐AIs are also naturally present in plants, where they act as defense chemicals from biotic stress (Barrett & Udani, 2011; Sun, Warren, & Gidley, 2019). Over 800 plant species have been reported to have antidiabetic activity (de Sales et al., 2012). In particular, alkaloids, polysaccharides, steroids, glycopeptides, terpenoids, and several polyphenols (including hydroxycinnamic acids, chalcones, epicatechin, flavans, and anthocyanins, as shown in Figure 2, have been described as possible low‐MW drug candidates (Kaeswurm, Claasen, Fischer, & Buchweitz, 2019; Kaur et al., 2021; Li, Bai, Jin, & Svensson, 2022; Papoutsis et al., 2021).

Among natural inhibitors, however, much attention has been paid to certain proteins which interact with α‐amylase, thus providing a protein–protein interaction capable of reducing hydrolyzing activity. Proteinaceous α‐AIs can derive from microbial and plants sources (Li, Fan, & Zhao, 2022). The latter can be further divided into six different classes: the knottin‐like type, the Kunitz type, the thaumatin‐like type, the γ‐thionin‐like type, the cereal type, and the lectin‐like type (recently well reviewed in Li et al., 2021). The first three classes only affect the amylases insects, and can be therefore used in agriculture as biological pest control methods.

However, some classes can interact with mammalian (and human) enzymes and thus represent a potential drug candidate for antidiabetic and overweight treatments. The greatest interest has been addressed toward legume lectin‐like glycoproteins extracted from several legumes (Bharadwaj, Raju, & Chandrashekharaiah, 2018; Yamada, Hattori, & Ishimoto, 2001). In particular, the commercial proprietary PHASE 2® is produced from the common bean *Phaseolus vulgaris*, as well as other similar “carb controller” products (Barrett & Udani, 2011; Ramírez‐jiménez, Reynoso‐camacho, Tejero, León‐Galván, & Loarca‐Piña, 2015; Udani et al., 2018).

In this work, we review the recent Literature about their structure, mechanism of action and clinical efficacy, especially focusing in the last 10 years papers and clinical meta‐analysis.

## 2 AMYLASE INHIBITORS FOUND IN *P. vulgaris*

Among the described α‐AIs, the proteinaceous ones from *P. vulgaris* L. (α‐AIs) show a particularly high potential (Bosi et al., 2019). In fact, the common bean is a well‐widespread cultivation worldwide, with several positive nutritional features. Besides, these inhibitors are generally recognized as safe (no side effects found, as for other plant inhibitors; Kusaba‐Nakayama et al., 2000).

α‐Amylase inhibitors are lectins (carbohydrate‐binding proteins, causing agglutination or precipitation of cells, glycoconjugates, including glycoproteins). *P. vulgaris* expresses three main classes of lectins: α‐AIs, phytohemoagglutinins (PHA), and arcelins (ARL), as protection agents of seeds from biotic (i.e., insect predation) and abiotic stress (i.e., drought, salinity, and wounding) (Mojica & de Mejía, 2015; Moreno, Altabella & Chrispeels, 1990), all deriving from a single ancestral gene (Kluh et al., 2005). PHA is a mitogen, affecting the intestinal mucosa of mammals, whereas ARL prevent absorption of nutrients in insect larvae (Santimone et al., 2004). These components are regarded as antinutritional factors (Obiro, Zhang, & Jiang, 2008), but thermal denaturation (typically reached during cooking) inactivates anti‐nutrient agents, adding nutritional value due to their high sulfur aminoacid content (Mojica & de Mejía, 2015).

Three main α‐AIs can be found in common beans: isoform 1 (α‐AI1), isoform 2 (α‐AI2), and α‐amylase inhibitor‐like (α‐AIL) (Ishimoto, Suzuki, Iwanaga, Kikuchi, & Kitamura, 1995; Obiro et al., 2008). All these proteins are specifically able to inhibit animal α‐amylases, not affecting plant amylases (Moreno, Altabella, & Chrispeels, 1990). Due to their activity against insect amylases, these proteins have been proposed as insecticides against bruchid pests (Le Berre‐Anton, Nahoum, Payan, & Rougé, 2000). In fact, their genes have been also transgenically expressed in other plants (such as tobacco and peas) to confer resistance to various insect pests (Altabella & Chrispeels, 1990; Schroeder et al., 1995).

On the other hand, only α‐AI1 has a specific effect on mammalian (and thus human) amylases, being therefore the target for nutraceuticals clinical studies. Heterologous expression of α‐AI1 in yeasts has also been recently described to improve potential large‐scale production (Brain‐Isasi, Álvarez‐Lueje, & Higgins, 2017).

### 2.1 Amylase inhibitors genes

As aforementioned, seven types of natural protein inhibitors of α‐amylases (α‐AIs) have been identified, and six of these, namely the knottin‐like type (Bhide et al., 2017), the γ‐thionin‐like type (Mojica, de Mejia, Granados‐Silvestre, & Menjivar, 2017), the cereal type, the Kunitz type, the thaumatin‐like type, and the lectin‐like type (Richardson, 1991), are extracted from plants. The other type has been isolated from bacteria (Sumitani, Tsujimoto, Kawaguchi, & Arai, 2000). The molecular weights of these inhibitors range from 3 to 23 kDa (Bhide et al., 2017). The knottin‐like type, the Kunitz‐type, and the thaumatin‐like type are able to inhibit amylase only in insects (Pereira et al., 1999).

The α‐AI lectin‐like glycoproteins have been isolated from legumes. They were named for their high degree of amino acid sequence homology compared with the lectin proteins abundantly expressed in these plants.

The currently known α‐AI isoforms present in the different cultivars of the legume, *P. vulgaris* are α‐AI1 (Berre‐Anton, Bompard‐gilles, & Rouge, 1997; Hoffman & Donaldson, 1985), α‐AI2 (Suzuki, Ishimoto, & Kitamura, 1994), α‐AIL (Finardi‐Filho, Mirkov, & Chrispeels, 1996), α‐AI4, and α‐AI5 (Lee, Gepts, & Whitaker, 2002). All the α‐AI isoforms are encoded by genes that are part of a gene cluster, which includes *dlec1* (encoding lectin phytohemagglutin PHA‐E), *dlec2* (encoding leukophytoagglutinin PHA‐L), and *Arc* (encoding arcelin) (Mirkov et al., 1994; Nodari, Tsail, Gilbertson, & Gepts, 1993).

According to evolutionary studies, the gene cluster may have originated following gene duplication events (Nodari et al., 1993). The gene cluster encodes proteins that share similar characteristics in their secondary and tertiary structures, as they are basically made up of antiparallel β‐sheets. ARL differ from PHA‐E and PHA‐L through the absence of a short amino acid sequence (gap deletion G3), which, if present, forms a short loop. α‐AI1 and α‐AI2 differ from ARL through the absence of two sequences (gaps deletions G1 and G2), which, if present, form two other loops.

Ultimately, the proteins α‐AI1 and α‐AI2 have the shortest sequences due to the deletion gaps G1, G2, and G3. The α‐AIL protein (α‐amylase inhibitor‐like protein previously called α‐AI3) differs from α‐AI1 and α‐AI2 mainly due to the insertion of 15 amino acids around position 115 (G2) of the primary structure (Finardi‐Filho et al., 1996).

**FIGURE 3:**
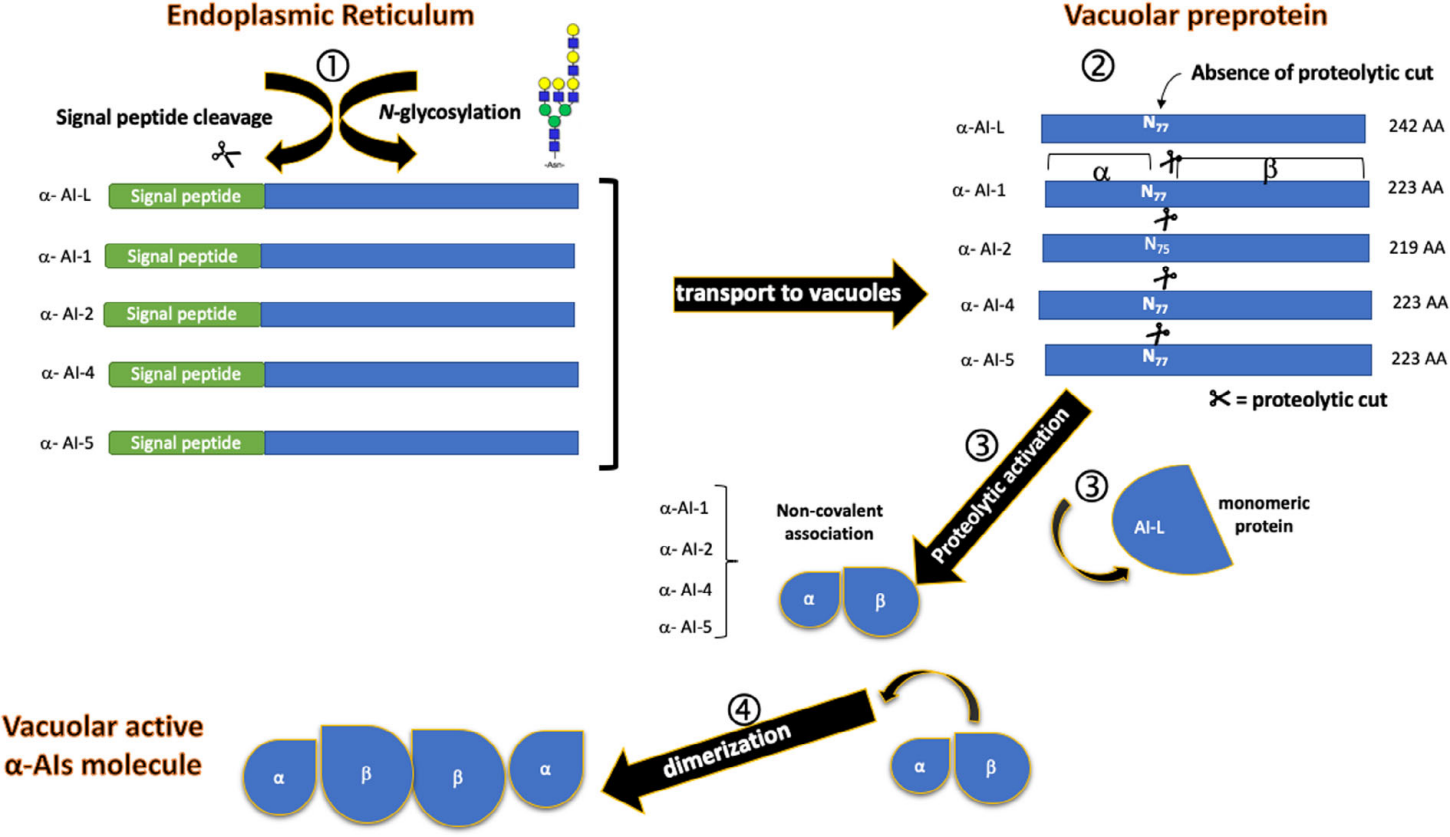
Different α‐AIs isoforms present different susceptibility to proteolytic activation. α‐AI, α‐amylase inhibitor

The α‐AIs in the bean, similar to the other proteins encoded by the gene cluster, are synthesized in the endoplasmic reticulum (ER) as inactive preproproteins to become active proteins once transported to the vacuoles. The transition from inactive to active protein involves several post‐translational modifications by which the precursor is *N*‐glycosylated and cut (except in the case of the α‐AIL isoform) at a conserved Asn residue to give rise to two subunits (called α and β) that assemble non‐covalently (Figure 3) (Berre‐Anton et al., 1997; Campbell et al., 2011; Moreno et al., 1990; Pueyo, Hunt, & Chrispeels, 1993; Yamaguchi, 1991).

**FIGURE 4:**
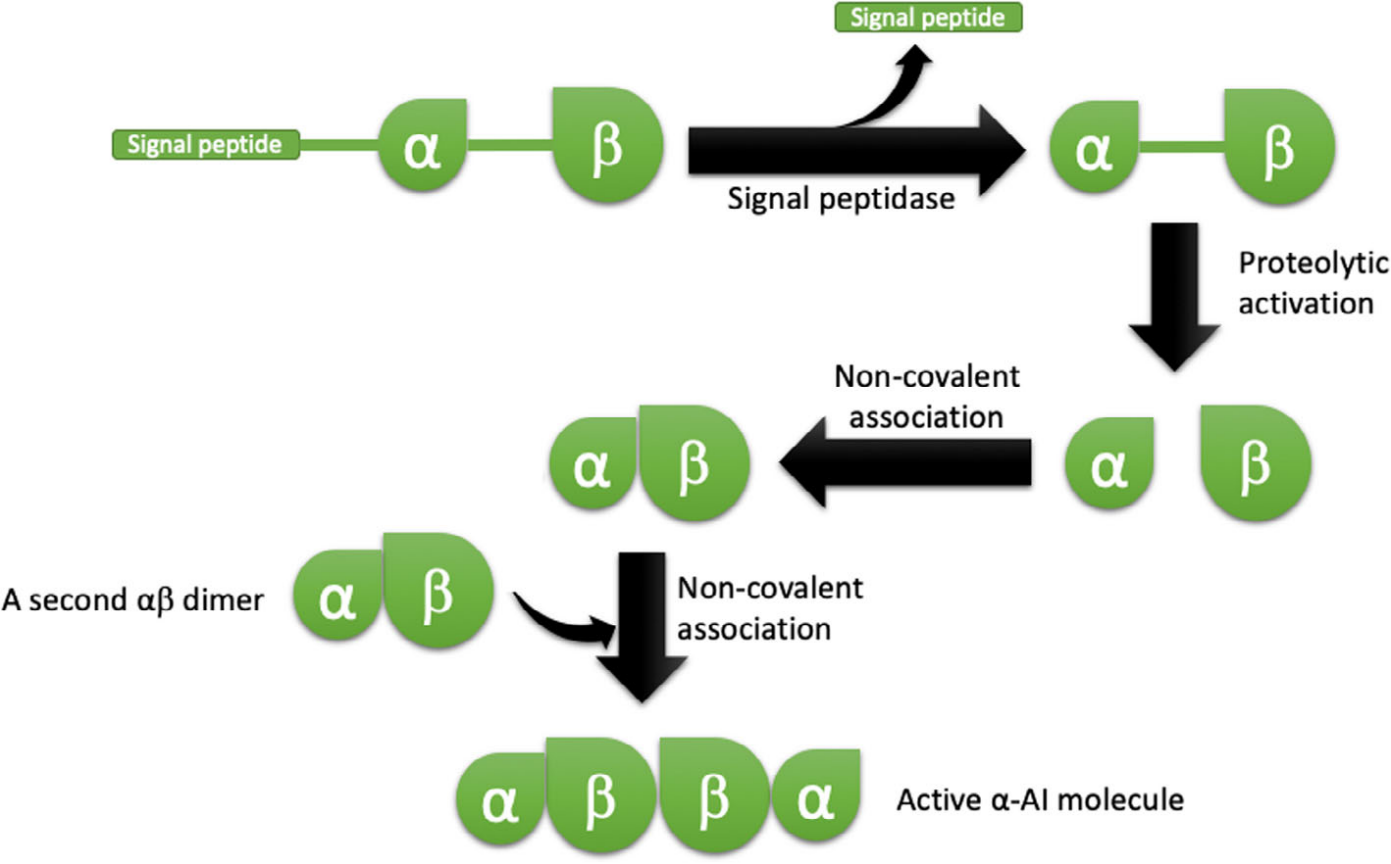
Post‐translational modifications of α‐AI1, α‐AI2, α‐AI4, and α‐AI5. Proteolytic activation includes the removal of signal peptide, and the proteolytic scission of subunits α and β. Then, dimerization leads to αβ dimers that associate in pairs to form the heterotetramer α_2_β_2_, the active α‐AI molecule (adapted from Lee et al., 2002). α‐AI, α‐amylase inhibitor

The α‐AIs of *P. vulgaris* generally show only small differences in their primary structures and in their *N*‐glycosylation sites. αAI‐1 was the first isoform of *P. vulgaris* to be isolated in 1945 (Bowman, 1945), molecularly characterized (Hoffman, Ma, & Barker, 1982; Moreno et al., 1990) and crystallized (Bompard‐Gilles, Rousseau, Rougé, & Payan, 1996). In the ER, the preprotein undergoes cleavage of the signal peptide and *N*‐glycosylated sites on residues of Asn12, Asn65, and Asn140 (the numbers refer to the positions of the residues in the mature protein). Through the secretory pathway, the protein is transported to the vacuoles, where it undergoes a second proteolytic cut. Specifically, vacuolar proteases cut at the conserved Asn77 residue (the number indicates the position of the residue after removal of the signal peptide) and also at the Asn223 to remove the C‐terminal portion, which is then used to direct the peptide transport to the vacuoles. These post‐translational modifications give rise to the α and β subunits, with molecular weights of 7,800 Da (77 amino acid residues) and 14,600 Da (146 amino acid residues), respectively (Moreno & Chrispeels, 1989; Yamaguchi, 1991). As shown in Figure 4, in the native protein, the protein subunits are then organized in a tetrameric structure α_2_β_2_ (Lee et al., 2002; Mirkov et al., 1994).

Site‐specific mutagenesis experiments have demonstrated the importance of the amino acid residue in position 77 of the mature protein for the acquisition of the native structure. In fact, the removal of the proteolytic cleavage site at Asn77 by substitution of the ATT codon with a GAT codon (Asn77→Asp77) in the gene α‐AI1 encoding the α‐AI1 isoform leads to the expression of an inactive inhibitor (Pueyo et al., 1993). In the αAI‐2 isoform, the proteolytic cleavage that gives rise to the α and β chains occurs at Asn75. This isoform has an α subunit that is shorter, by two amino acids, than the α subunit of α‐AI1 (Ser56 and Tyr57 are missing). It is interesting to note that α‐AIL, while retaining Asn77 in its primary structure, does not undergo proteolytic cleavage, so it is a monomeric protein (Finardi‐Filho et al., 1996).

**FIGURE 5:**
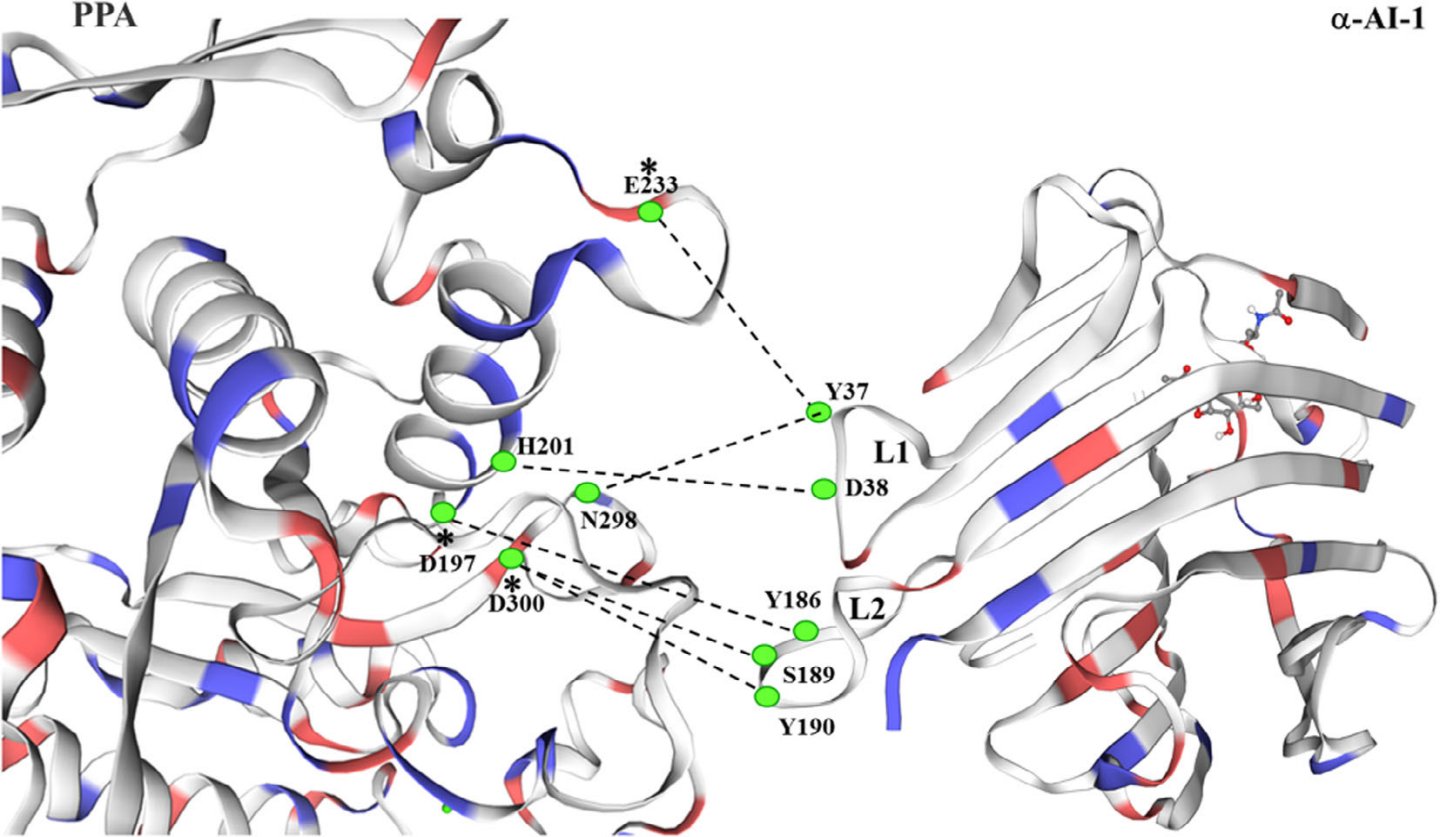
Three‐dimensional model structure of the PPA/α–AI1 complex. One monomer unit of α‐AI1 (template 1dhk.1.B) in interaction with a molecule of PPA (template 3l2l.1.A) is shown. The two hairpin loops (L1 and L2) of α‐AI1 interacting directly with the active site of PPA are highlighted. The α‐AI1 tyrosine, aspartate, and serine sidechains (Y37, Y186, Y190, D38, S189), important to the interaction with catalytically important residues of PPA enzyme (E233, D197, D300, H201, N298) are reported with the one‐letter code. The * indicate the three amino acids of α‐AI1 directly involved in the catalytic mechanism. The 3D structures were generated using SWISS‐MODEL (http://swissmodel.expasy.org) server. α‐AI, α‐amylase inhibitor; PPA, porcine pancreatic α‐amylase

By exploiting the ability of the protein isoform, α‐AI1, to interact with mammalian α‐amylases, Bompard‐Gilles and coworkers crystallized the α‐AI1–PPA complex (porcine pancreatic α‐amylase‐α‐AI1) in order to define the interaction domains that are established between the two proteins (Bompard‐Gilles et al., 1996). Contrary to the results obtained in previous studies (Marshall & Lauda, 1975), the analysis of the crystals showed that α‐AI1 consists of two dimers, and each is able to interact with a pancreatic enzyme molecule, PPA. Its secondary structure is devoid of α‐helices and contains an abundance of β‐antiparallel sheet. In the inhibition process promoted by αAI‐1, two hairpin loops in the inhibitor (L1 and L2) play an important role. The amino acid residues in the loop make direct and indirect hydrogen bonds via H_2_O molecules with the enzyme (see below). L1 includes residues 29–46, and L2 includes residues 171–190. The inhibitor–enzyme interactions lead to conformational modifications of α‐AI1 projecting the Tyr37 and Asp38 residues of L1 and the Tyr186 and Tyr190 residues of L2 into the active site of the enzyme, where three amino acid residues directly involved in acid–base catalysis events are present (Asp197, Glu233, and Asp300, as shown in Figure 5). Tyr37 of the inhibitor, as a result of conformational changes induced by interactions with the enzyme, forms hydrogen bonds with the Glu233 residue and part of the Asp300 residue present in the active site of the enzyme. The latter is also engaged in binding with a chloride ion. The flanking Asp38 residue of the inhibitor assumes a position favorable for the formation of a hydrogen bond with His201, which is also present in the active site of the enzyme. The formation of this hydrogen bond facilitates the interaction of Tyr186 with the enzyme, which is projected into the heart of the catalytic site, forming hydrogen bonds with the catalytic nucleophile, Asp197 (McCarter & Withers, 1996). The orientation of the L2 loop allows the formation of a hydrogen bond with the Asp197 residue of the enzyme, which will not be able to participate in the catalytic event as a nucleophile (McCarter & Withers, 1996). Asp300 in the enzyme, the third residue of the catalytic triad involved in the acid–base catalysis with the substrate, is blocked, as it forms two hydrogen bonds with the inhibitor: the first bond is established between the carboxyl of its side chain and the hydroxyl group of Ser189 in the inhibitor, and the second bond is established between its carbonyl oxygen and Tyr190 in the inhibitor.

Additional molecular information on *P. vulgaris* α‐AIs was obtained after the isolation of the genes encoding the protein isoforms α‐AI2 and α‐AIL (Finardi‐Filho et al., 1996; Mirkov et al., 1994; Suzuki et al., 1994). The reconstruction of the primary structure of the two proteins allowed to define their hypothetical three‐dimensional structures using the crystal structure obtained by Bompard‐Gilles et al. (1996) for the PPA–α‐AI1 complex as a model. Despite the high degree of similarity of the three isoforms, α‐AI1, α‐AI2, and α‐AIL, their specificities to different α‐amylase enzymes vary. While α‐AI‐1 inhibits the activity of pig α‐amylases and those of the insects *Tenebrio molitor*, *Callosobruchus maculatus* and *Callosobruchus chinensis*, the other two isoforms do not act on these enzymes. α‐AI2 inhibits the α‐amylase of the *Zabrotes subfasciatus* beetle (ZSA), while α‐AI1 does not. To explain whether these differences could be justified at the molecular level, the two isoforms, α‐AI2 and α‐AIL, were modeled in three dimensions using PPA/α‐AI1 and PHA‐L crystals as models (Hamelryck et al., 1996). Although all the isoforms had very similar three‐dimensional structures, both α‐AI2 and α‐AIL differed from α‐AI1 by some more or less extensive structural changes. The results of the crystallographic studies obtained by Bompard‐Gilles et al. (1996) confirmed, in detail, the hypotheses on the structural differences between α‐AI1, α‐AI2, and α‐AIL formulated by Finardi‐Filho et al. (1996). Similar to α‐AI1, the α‐AI2 isoform is a glycoprotein consisting of two chains resulting from the proteolytic processing of a preprotein on an asparagine residue (Asn75) equivalent to the Asn77 of α‐AI1. The proteolytic cleavage of Asn75 occurs due to the deletion of two amino acid residues at the *N*‐terminus of the α chain (Tyr34 and Asn35). Other molecular differences include the replacement of the Tyr37, Tyr186, and Tyr190 residues present in α‐AI1 with Val35, His175, and Phe179 in α‐AI2. The α‐AI2/PPA docking experiments revealed a binding pattern between α‐AI2 and PPA similar to that found for the α‐AI1–PPA complex. However, the deletion of the two amino acids and the amino acid substitutions in α‐AI2 result in much weaker α‐AI2–PPA interactions than those described for the α‐AI1–PPA complex. In the α‐AI2–PPA complex, 8 hydrogen bonds are established compared to the 15 hydrogen bonds in the α‐AI1–PPA complex. These weak interactions justify the inability of α‐AI2 to inhibit PPA. Although their overall three‐dimensional structures are very similar, α‐AIL differs from α‐AI1 or α‐AI2 as a result of two extra loops of 15 (stretch 95–109) and 6 (stretch 121–126) residues that are absent in α‐AI1 and α‐AI2. Furthermore, α‐AIL, unlike the two other inhibitors, does not undergo any proteolytic cleavage, as shown in Figure 3; it is a single‐chain protein. The docking experiments performed with PPA show that, based on these structural differences, α‐AIL cannot bind to the catalytic site of enzymes PPA and ZSA.

The docking experiments conducted using the α‐AI2 isoform and the ZSA enzyme have highlighted the establishment of 12 hydrogen bonds compared with only 8 hydrogen bonds that are formed in the α‐AI1–ZSA complex. Since ZSA is inhibited only by α‐AI2 (Grossi de Sa et al., 1997), the network of 12 hydrogen bonds that is established between ZSA and α‐AI2 is sufficient to stabilize the complex. Conversely, the network of eight hydrogen bonds observed between α‐AI1 and ZSA would not be strong enough to stabilize this complex, and this may explain why α‐AI1 does not inhibit ZSA.

The information obtained during the molecular studies conducted on the α‐AI isoforms was exploited with the aim of highlighting additional coding genes and therefore other protein isoforms of the inhibitor. Chrispeels and Raikhel (1991) reported that these genes encode proteins highly conserved in the amino (presence of MASS^K^/_N_ residues) and carboxyl terminals (LN^Q^/_K_IL residues), suggesting the importance of the sequences for synthesis in the ER and transport to the vacuole. Using pairs of primers on the nucleotide regions with maximum amino and carboxyl terminal homology, researchers performed PCR experiments to analyze DNA extracted from cultivar WKB 858 and black bean (BB) cultivar of *P. vulgaris* (Lee et al., 2002). The sequencing of the PCR products allowed the identification of two genes (α‐AI4 for the 858 cultivar and α‐AI5 for the BB cultivar) highly homologous to the other genes coding for the α‐AIs. The primary structures of the two isoforms deduced from the nucleotide sequences of α‐AI4 and α‐AI5 were compared with those of the isoforms α‐AI1, α‐AI2, α‐AIL (homology 93–77%), and with those of PHA‐E, PHA‐L, and ARL proteins (homology 55–40%). The high‐sequence homology strongly confirmed that these genes evolved from a common ancestral gene (Lee et al., 2002). The primary structures of the two isoforms, α‐AI4 and α‐AI5, were similar to those of the other isoforms. They all had three *N*‐glycosylation sites and the recognition sequence for the peptidase that cuts the link between Ser21 and Ala22. The two isoforms also had the recognition sequence for the vacuolar protease that cuts the bond between Asn77 and Ser78, in line with what has been reported for the α‐AI1 and α‐AI2 isoforms.

The interaction between α‐AI1 and amylases seems to happen in the same site of acarbose (Payan, 2004; Qian, Haser, Buisson, Duee, & Payan, 1994).

The three‐dimensional structures of these two isoforms, based on the information obtained from the crystallographic studies of the α‐AI1–PPA complex, highlight the presence of the three residues (Arg74, Trp188, Tyr190) important for the inhibition of α‐amylase. In particular, it has been suggested that the active inhibitor site consists of Trp188 (β subunit), Arg74 (α subunit), and Tyr190 (β subunit), resembling the Trp‐Arg‐Tyr motif of bacterial α‐AI tendamistat (Mirkov et al., 1995). In this perspective, the proteolytic activation at Asn77 may be necessary to bring these residues in close proximity. Besides, two tyrosines (Tyr37 and Tyr186) from the two hairpin α‐AI1 loops interact with two key residues in the enzyme active Asp197 and Glu233 (Payan, 2004). These two nucleophilic and acid functions act as crucial catalysts in the amylase action.

Besides, further protein–protein interactions involve areas away from the active site, including the loop 303–312, the loop at position 237–240, the loop 347–357, and the loop 140–150 from domain B (Payan, 2004). However, some differences in α‐AI1 inhibition have been observed between human and pig pancreatic amylases, being possibly due to the aminoacidic sequence dissimilarity in the two enzymes (Nahoum et al., 2000). For instance, some additional hydrogen bonds between inhibitor and human enzyme domain B have been observed, as well as different patterns of interactions in the loop regions 303–312 and 347–357 of domain A (Payan, 2004).

On the whole, this complex network of protein–protein interactions mimics the substrate binding in several subsites of the enzyme structure, inducing a steric hindrance process blocking the access of starch to the active site, which prevents hydrolysis. However, this process seems to be different if compared with the action of carbohydrate inhibitor acarbose. In this case, conformational changes have been reported in the flexible loop (residues 303–309), in the loop 237–240, and in the segment at position 140–150 of the domain B (Payan, 2004; Qian et al., 1994). The main residues involved are the catalytic residue Asp300 and residue His305, which undergo deep conformational changes. After substrate binding, the flexible loop moves toward the inhibitor, leading to a reduction of the cleft breadth. On the contrary, when α‐AI1 binds the enzyme, a tight‐binding process occurs, pushing away the same loop toward the solvent. The loop region 351–359 in domain A undergoes significant structural modifications, while Asp 300 does not modify the orientation if compared with free amylase.

However, the process of α‐AI1 has not been completely elucidated, being the role of some residues still not completely and universally understood (Obiro et al., 2008). This should encourage further studies, in perspective of the development of more (semi)synthetic inhibitors (see below).

All the α‐AI isoforms, despite having a high‐sequence homology overall, differ consistently in their upstream and downstream regions, which contain the amino acid residues responsible for the formation of hydrogen bonds with the active site of α‐amylases (Lee et al., 2002). It is probable that the low‐sequence homology in this region is responsible for each isoform's specificity for α‐amylases. In fact, studies on the α‐AI1 and α‐AI2 isoforms have shown that the differences in these regions lead to a different orientation of the L1 and L2 hairpin loops when the inhibitor comes into contact with the active site of the enzyme. Knowing the structural details of this family of plant α‐AIs and their mechanism of action could help to understand their important roles in the control of endogenous amylases and in the protection against pathogens and parasites. Such pieces of information could also enhance the development of new inhibitors resembling the mechanism of α‐AIs.

### 2.2 Structural properties

In the common bean, α‐AI is a 43 kDa‐residue polypeptide with three disulfide bridges that includes different isoforms (see above) (Berre‐Anton et al., 1997; Pallaghy, Norton, Nielsen, & Craik, 1994). As stated above, the active sites of amylases usually comprise three key amino acids, namely Asp197, Glu233, and Asp300 (Brayer, Luo, & Withers, 1995). The α‐amylase‐binding site could hold at least six monosaccharide structures and be cleaved between the third and fourth pyranose residues by a double displacement mechanism (Pereira et al., 1999).

The inhibitor α‐AI1 is purified from a variety of kidney bean seeds and is a 43 kDa tetrameric glycoprotein (α_2_β_2_) with a Stokes radius of 29.0 Å, which corresponds to globular proteins. Chemical and enzymatic deglycosylation methods gave *M*_r_ calculated from sodium dodecyl sulfate–polyacrylamide gel electrophoresis (PAGE) experiments of about 7.8 and 14 kDa for α and β subunits, respectively (Yamaguchi, 1991). All these protein fractions react with biotinylated ConA, thus indicating they are glycosylated. The branched glycans linked to Asn65 and Asn140 of α‐AI1 residues, protrude in the solvent at the back face of the dimer (Santimone et al., 2004).

α‐AI1 has in turn wo isoforms in *P. vulgaris* cv Tendergreen, α‐AI1 and α‐AI1′, which differ from each other by their isoelectric points and neutral sugar contents (Berre‐Anton et al., 1997; Kasahara et al., 1996). Both α‐AI1 and α‐AI1′ give a single protein band when analyzed by PAGE in native conditions, with an estimated *M*_r_ of 43.6 and 39.8 kDa, respectively (Berre‐Anton et al., 1997). The yield of neutral sugar contents of α‐AI1 and α‐AI1′ is 16% and 14% (*w/w*) respectively as estimated according to the phenol–H_2_SO_4_ method (Dubois, Gilles, Hamilton, Rebers, & Smith, 1956). According to the vapor phase chromatography, the oligosaccharide chains of both inhibitors are mainly composed of mannose associated to a trace of xylose (Yamaguchi, Funaoka, & Iwamoto, 1992). α‐AI1 is a lectin‐like amylase non‐competitive inhibitor that has been found both in mammalian and two kinds of insects' amylases that come from *C. maculatus* and *C. chinensis* (Li et al., 2021).

The occurrence of two extra‐loops on the front face of α‐AIs prevents these proteins from entering the substrate cleft of PPA; thus, the removal of these loops appears as a structural requirement for the α‐amylase inhibitory activity of α‐AIs. In addition, as discussed above (Figure 4), the proteolytic processing occurring at Asn77 residue, which cleaves the polypeptide chain of α‐AI1 in an α‐chain (residues 1–77) and in a β‐chain (residues 78–223), is necessary to transform an inactive precursor in a fully active inhibitor (Pueyo et al., 1993).

Several studies confirmed that α‐AI1 interacts with a large region that surrounds the substrate‐binding site of PPA in a slow process and forms a 1:2 stoichiometric complex which exhibited an optimum pH of 4.5 at 30°C (Berre‐Anton et al., 1997; Franco, Rigden, Melo, & Grossi‐de‐Sá, 2002). The total buried area contributed by both molecules in this complex is about 3,050 Å^2^, which is reported to be one of the largest values for a protein–protein complex (Bompard‐Gilles et al., 1996). α‐AI1 was also similarly shown to interact with the *T. molitor* α‐amylase (Virginie Nahoum et al., 1999).

### 2.3 Operational stability

Several factors affect α‐AI activity, including pH, temperature, and several ions. While mammalian amylase presents the highest activity close to neutrality (6.5–7), the optimum of pH for inhibitory activity has been identified in a slightly acidic environment, in the range 4.5–5.5 (Kotaru et al., 1987; Lajolo & Filho, 1985; Marshall & Lauda, 1975; Powers & Whitaker, 1978), being almost inactive outside this range (Berre‐Anton et al., 1997). Lajolo and Finhardi Filho highlighted a specific difference between salivary (4.5) and pancreatic amylases (5.5) (Lajolo & Filho, 1985). Other authors showed an optimum at neutrality using porcine pancreatic amylase, but no incubation was performed in this case (Gibbs & Alli, 1998). Similar values of pH (5.25) have been also shown to positively affect inhibitor purification (Wang, Chen, Jeng, & Sung, 2011).

In fact, incubation time seems to greatly affect inhibitory activity, possibly explaining the different pH optima observed. A period of time comprised between 10′ and 120′ has been used to reach optimum activity (Berre‐Anton et al., 1997; Marshall & Lauda, 1975; Powers & Whitaker, 1978): shorter incubation was in fact necessary when operating at pH 4.5. These findings suggest that, to obtain in vivo effects, nutraceutic preparations based on α‐AI should be ingested before the meals, or at least at the same time (Obiro et al., 2008). The pre‐incubation pH could also enhance α‐AI effects. For instance, 20′ at pH 4 led to a boost of inhibition (Kluh et al., 2005), even though these data were collected on insect amylases.

Incubation times and pH seem to interfere also when the temperature effect on α‐AI is evaluated. In fact, when operating at pH 4.5, a maximum of activity between 22 and 37°C was observed, with no significant differences within this range (Berre‐Anton et al., 1997). Lajolo and Filho (1985) showed only a slight increase in activity in the range 25–45°C, incubating inhibitor and enzymes at pH 5.4. On the contrary, a 10‐fold increase in activity has been detected pre‐incubating the inhibitor at amylase optimum pH (6.9) (Marshall & Lauda, 1975). These findings could be explained by the lower energy barrier at acidic pHs (Lajolo & Filho, 1985). All studies agree on the complete loss of activity boiling at 100°C and refrigerating at 0°C (Berre‐Anton et al., 1997; Marshall & Lauda, 1975; Valencia, Bustillo, Ossa, & Chrispeels, 2000). In a study on transgenic peas, however, α‐AI retained almost all the inhibitory effect when treated for 5′ at 80°C (an almost complete drop in activity was observed for higher temperatures) (Collins, Eason, Dunshea, Higgins, & King, 2006).

Several ions also affect the binding between α‐AI and amylase, and the consequent inhibitory effect. Particularly, calcium ion (5 mM) is important for the rate of initial binding (Gibbs & Alli, 1998). Chloride ion is also necessary to reach optimum activity (Gibbs & Alli, 1998; Lajolo & Filho, 1985). In fact, both these ions are usually present in the typical protocol commonly used for the determination of inhibitory activity. Potassium, magnesium, and sulfate ions did not interfere with α‐AI biological activity (Gibbs & Alli, 1998), whereas a positive effect of nitrate, bromide, iodide, and thiocyanate ions has been described (Lajolo & Filho, 1985).

Taken together, all these data show several discrepancies about the optimal operational conditions for inhibition activity. This suggests the need of further studies to evaluate more carefully the interactions between pH, temperature, and incubation times, and to identify more precisely the best conditions for processing and administration of possible α‐AI‐based preparations.

## 3 CLINICAL TRIALS

According to WHO, obesity is defined as abnormal or excessive fat accumulation. The body mass index (BMI), calculated by dividing a person's weight in kilogram by the square of height in meters (kg/m^2^), represents a measure of obesity: a person is considered obese with a BMI ≥30.

The cause of excess body weight is an imbalance between energy intake and expenditure.

The WHO identifies two main factors which can cause the obesity condition: on one side the increasing intake of food rich in fat and sugar but lacking in vitamins and minerals, and on the other the decrease in physical activity due to an increase in sedentary works, urbanization, and motorized transports (WHO, 2021).

As mentioned above, many in vivo studies have shown that extracts of *P. vulgaris* have anti‐obesity effects in animals and human models, presenting several advantages if compared to traditional dietary or pharmacological interventions (Bosi et al., 2019; Kusaba‐Nakayama et al., 2000). On the contrary, in vitro models have not been usually employed. In fact, the overall effect on blood glucose of α‐amylase:α‐AI interaction can be difficulty observed by such models. So, in this review we focused only on in vivo studies.

### 3.1 *In vivo* studies on animal models

Many studies related to the administration of α‐AI extracts to rats have been collected in a paper review by Carai et al. (2009). Unfortunately, the different ways used to express enzyme and/or inhibitor activities in units rather than in absolute weight terms make comparisons not easy to obtain.

The inhibition of the growth rate in rats fed with raw *P. vulgaris* beans and extract changes biochemical parameters and alteration in organ histology, as reported since the 1960s (Kakade & Evans, 1966; Maranesi, Barzanti, Biagi, Carenini, & Gentili, 1984; Maranesi, Carenini, & Gentili, 1984). Only a transitory pancreatic enlargement was observed in young healthy rats consuming raw kidney beans chronically, accompanied by a significant reduction in body content of lipid and a lower body weight gain compared with control group (Grant, Dorward, Buchan, Armour, & Pusztai, 1995).

A weight loss (WL) was also observed after the administration of purified α‐AI for 10 days, but it was accompanied by intestinal blockage (cecum level) by solidified digesta in healthy Lister Hodded rats, since starch digestion was almost completely blocked (Pusztai et al., 1995). This effect, recorded only when the highest inhibitor concentrations (20 and 40 mg/kg) were administered, was linked to bacterial fermentation causing the hypertrophic growth of the tissue leading in some cases to the organ rupture.

On the contrary, using a different dry *P. vulgaris* extract, the administration of various doses (0, 50, 200, and 500 mg/kg) for 10 days did not imply side effects, but a reduction in rat body weight associated with a decrease in food intake was observed (Fantini et al., 2009).

This anorexigenic effect, observed by many authors (Loi et al., 2013; Shi et al., 2020; Tormo, Gil‐Exojo, de Tejada, & Campillo, 2004; Tormo, Gil‐Exojo, de Tejada, & Campillo, 2006), may be related to the lectin activity on the intestinal brush border, by altering the release of cholecystokinin and glucagon‐like peptides that play an important role in the control of appetite. This phenomenon has been observed also when high palatable food was administrated to rats (Carai et al., 2009; Fantini et al., 2009).

It has been demonstrated that *P. vulgaris* extract is also effective in reducing glycemia in healthy and diabetic rats (Tormo et al., 2004; Tormo et al., 2006), as well as glucose absorption.

It is interesting that the antihyperglycemic activity is more effective if the *P. vulgaris* seeds are mixed with glibenclamide, an oral hypoglycemic agent. It was demonstrated that the combination of 300 g/kg (weight of seeds per body weight) with 0.20 g/kg body weight of gilbenclamide results in a safer and potent hypoglycemic and antihyperglycemic activity in chronic diabetic conditions (Ocho‐Anin Atchibri, Brou, Kouakou, Kouadio, & Gnakri, 2010).

Different results were obtained when crude white bean flour was administrated (1 g/kg body weight) to diabetic‐induced Wistar rats for 21 days; no significant decrease in blood glucose was recorded in this case (Pereira et al., 2012).

These different results could be linked to the different degree of purification of the extract in accordance with what observed in human model (see below) to various dosages and to the absence of a joint action with hypoglycemic agents.

Similarly, no significant WL, change in organ weight, food consumption, and blood biochemical parameters were recorded also in healthy Sprague–Dawley rats that received white kidney bean extract at doses 4, 2, and 1 g/kg for 90 days (Qin et al., 2019).

Qin et al. (2019) suggest that this difference could be related to the experimental group state of health. Healthy rats are in fact probably more resistant to α‐AI effect compared with obese rats. Previous studies showed that amylase activity is higher in healthy subjects than in obese subjects (both rats and humans) (Kondo, Hayakawa, Shibata, Sato, & Toda, 1988; Schneeman, Inman, & Stern, 1983), and so the inhibitor could act more effectively on healthy individuals.

The studies on animal models (as summarized in Table 1) have shown the potential of *P. vulgaris* extract as anorexigenic and antihyperglycemic agent although these properties depend on the type of extract administered, the duration of the trial, and the health state of animals.

**TABLE 1:** Main studies on animal models to test the efficacy of *Phaseolus vulgaris* extract

Reference	Administration	Subjects	Side effects	Main results
Grant et al. (1995)	Raw beans	Healthy rats	Transitory pancreatic enlargement	Reduction in body lipid level
Pusztai et al. (1995)	Partially purified extract	Healthy rats	Intestinal blockage (cecum level) by solidified digesta	Weight loss
Fantini et al. (2009)	Dry extract	Healthy rats	No side effects	Reduction in rat body weight, glycemia and a decrease in food intake
Tormo et al. (2006)	Purified extract	Healthy and diabetic rats	No side effects	Reduction in glycemia and decrease in food intake in both group
Loi et al. (2013)	Partially purified extract	Healthy rats	Not available	Reduction in normal and palatable food intake ang glycemia
Tormo et al. (2004)	Purified extract	Healthy rats	No side effects	Anoressigenic effect, reduction in weight gain, and blood glucose level
Ocho‐Anin Atchibri et al. (2010)	*P. vulgaris* seeds + gilbenclamide	Diabetic rats	No side effects	Potent hypoglycemic and antihyperglycemic activity
Pereira et al. (2012)	Bean flour crude	Diabetic rats	No side effects	No change in physiological parameters, in biochemical marker and organ weight
Qin et al. (2019)	Aqueous extract	Healthy rats	No side effects	No significant weight loss, change in organ weight, food consumption, and blood biochemical parameters

These results have laid the foundations for studying the role of beans extracts on human being with particular regard to the safety of the administered product.

### 3.2 Early studies on humans

The α‐AI from *P. vulgaris* powder has also been tested on human models.

Many of the early studies carried on in 70s and 80s on the bean extract inhibitor are discussed in different reviews (Barrett & Udani, 2011; Obiro et al., 2008; Udani, Hardy, & Kavoussi, 2007).

In the first studies, crude white bean‐based extracts were used. The results were disappointing; no decrease in human starch digestion was observed because of insufficient inhibitor activity and instability (Bo‐Linn, Ana, Morawski, & Fordtran, 1982; Carlson, Li, Bass, & Olsen, 1983; Hollenbeck et al., 1983).

A series of subsequent and more promising studies were published by a research group from the Mayo Clinic (Rochester, MN, USA) in the 1980s using a partially purified white bean product (Layer, Carlson, & Dimagno, 1985; Layer, Zinsmeister, & DiMagno, 1986).

In vitro it was found that the partially purified inhibitor inactivated intraduodenal, intraileal and salivary amylase without being affected by exposure to gastric juice (Layer et al., 1985).

These findings were also confirmed in vivo, by the reduction of amylase activity in the duodenal, jejunal, and ileal intralumen after the administration of partially purified amylase inhibitor accompanied by a significant reduction of gastric inhibitory polypeptide (GIP) concentration (Layer, Rizza, Zinsmeister, Carlson, & DiMagno, 1986).

These results were later confirmed by other authors who observed as the partially purified extract significantly reduced absorption of complex carbohydrates from the terminal ileum and decreased GIP concentration (Brugge & Rosenfeld, 1987; Jain et al., 1989).

In a series of studies, several doses of partially amylase inhibitor were administered to both healthy and diabetic subjects. A reduction in starch digestion and in postprandial glucose, C‐peptide, insulin, and GIP level was recorded (Boivin, Flourie, Rizza, Go, & DiMagno, 1988; Boivin, Zinsmeister, Go, & DiMagno, 1987). The only side effect was represented by a temporary diarrhea, and the authors suggested that impurities in the partially purified preparation may be responsible for this side effect.

Similar results were obtained by Layer, Rizza, et al. (1986), testing 13 subjects (eight healthy volunteers and five subjects with type 2 diabetes). The effect of a partially purified bean‐derived amylase inhibitor was recorded on the ingestion of 50 g of a test meal made of rice, cocoa, and aspartame. Also in this case, a decrease in postprandial plasma glucose and insulin concentration in both healthy and diabetics subjects was observed.

In 2000, the first study was conducted to investigate the effectiveness of white bean extract in WL (Thom, 2000).

An inhibitor product (Suco‐Bloc®) tablet (containing 200 mg of white kidney bean extract, 200 mg of inulin, and 50 mg of *Garcinia cambogia* extract) was administered to 40 overweight healthy subjects (BMI between 27.5 and 39).

In this randomized, double‐blind, placebo‐controlled trial the subjects had to eat two inhibitor tablets after all three meals per day for 12 weeks. After 3 months, a significative WL, a reduction of BMI, and a decrease of body fat percentage in the tested group were recorded. No sides effects were recorded in the experiments.

These papers confirmed the preliminary results obtained in the animal models. Not only the inhibitory activity is unaffected by gastric juices, but it reduces the absorption of complex carbohydrates and lowers blood glucose concentration, C‐peptide, and GIP levels, with almost negligible undesirable effects.

Moreover, the extract effectiveness in WL laid the groundwork for the study of commercial products that could be used as dietary supplements in the fight against obesity and diabetes. Further experiments became, in fact, necessary to improve purifications techniques and to test the safety of the product.

### 3.3 PHASE 2: Commercial products

Subsequent studies have focused on using the starch blocker IQP‐PV‐101, known commercially as PHASE 2, but previously named Phaseolamin 2250, because 1 g of the product blocked 2,250 starch calories (Preuss, 2009). It is also marketed globally under the Starchlite® and PhaseLite® brands (Grube, Chong, Chong, & Riede, 2014).

It is a dried aqueous extract from the common white bean *P. vulgaris* (Pharmachem Laboratories; Kearny, NJ, USA) produced from non‐GMO whole kidney beans and certified as gluten free. It is used as a dietary supplement (odorless and tasteless) in various forms, including powders, tablets, and capsules.

Each lot of PHASE 2 has at least 3,000 α‐amylase‐inhibiting units (AAIU) per gram when tested at a pH 6.8 using potato starch as substrate and pancreatin as enzyme source (Barrett & Udani, 2011).

As declared in the Evaluation of the Generally Recognized as Safe (GRAS) status of PHASE 2 white bean (*P. vulgaris*) extract, a typically recommended dose is 1–2 capsule containing each 500 mg of PHASE 2, taken three times a day during daily meals, or 1,500–1,300 mg per day (Nicolosi, Hughes, & Bechtel, 2007). Heat treatments inactivates the hemagglutinating power and trypsin activity, thus ensuring the product safety (Chokshi, 2007).

### 3.4 Meta‐analysis on clinical trials

Many clinical studies about PHASE 2 are collected in two meta‐analyses. The most recent has been proposed by Udani et al. (2018), investigating the effectiveness of PHASE 2 in reducing body weight and fat.

They only included in their analyses those studies that met the main requirements collectively termed PICOS (Population, Intervention, Comparison, Outcomes and Study) criteria: Participants had to be obese or overweight; Intervention had to be done with PHASE 2; the Control group had to be compared with the test group; Outcome measurements had to include measurements of body or fat mass; and Study design had to be based on double‐blind, placebo‐controlled parallel or crossover trial, randomized or open‐label studies.

Based on these characteristics, they selected 11 studies for meta‐analysis for WL with a total of 573 subjects, and 3 studies for meta‐analysis for fat loss with a total of 110 subjects.

They found that the intake of PHASE 2 resulted in a WL difference of 1.08 kg and in a fat reduction of 3.26 kg.

These conclusions partially contradict what was previously stated by Onakpoya, Aldaas, Terry, and Ernst (2011) in a previous meta‐analysis: in fact they did not find any significant difference in WL between treated and control group.

As suggested by Udani et al. (2018), this difference may be due to the choice to include in the Onakpoya meta‐analyses all studies on *P. vulgaris*, and not only on PHASE 2. These differences could have led to the inclusion of studies based on different partially purified preparations. However, also the meta‐analysis from Onakpoya et al. showed a significant effect on fat loss, confirming a sharper effect on this body parameter.

### 3.5 Studies on WL in humans

Many authors focused their attention on testing the effectiveness of PHASE 2 in the loss of body weight and body composition (as summarized in Table 2).

**TABLE 2:** Main clinical trials reported on humans for PHASE 2® preparations

Reference	Duration	Subjects	Dose of PHASE 2®	Main results	Trial type
Udani, Hardy, and Madsen (2004)	8 weeks	*n* = 39	1,500 mg twice a day	No significant difference in WL and triglyceride level	Randomized, double‐blind, placebo‐controlled
Udani and Singh (2007)	4 weeks	*n* = 25	1,000 mg twice a day	No significant difference between in WL and reducing waist size	Double‐blind, placebo controlled
Celleno, Tolaini, D'Amore, Perricone, and Preuss (2007)	4 weeks	*n* = 60	445 mg	Statistically significant difference in reducing BW, BMI, fat mass, adipose tissue thickness, and waist, hip, thigh circumferences. Lean body preserved	Randomized, double‐blinded, placebo‐controlled
Wu, Xu, Shen, Perricone, and Preuss (2010)	8 weeks	*n* = 101	2,000 mg	Statistically significant in reduction in BW and waist circumference. No significant change in hip circumference	Randomized, double‐blinded, placebo‐controlled
Grube et al. (2014)	12 weeks	*n* = 123	1,000 mg three times a day	Statistically significant difference in WL, waist circumference and BMI reduction	Randomized, double‐blinded, placebo‐controlled
Grube et al. (2014)	24 weeks	*n* = 49	1,000 mg three times a day	73.5% of WM	Randomized, double‐blinded, placebo‐controlled
Wang et al. (2020)	35 days	*n* = 120	2,400 mg	Statistically significant difference in BW, BMI, fat mass, adipose tissue thickness, and waist circumferences	Randomized, double‐blinded, placebo‐controlled

Abbreviations: BMI, body mass index; BW, body weight; WL, weight loss; WM, weight maintenance.

In a series of papers, Udani and coworkers investigated the role of PHASE 2 in decreasing body weight, triglyceride (TG) level, and reducing waist size.

They did not achieve a significant statistical difference between active and placebo group in WL, by lowering TG level and reducing waist size, but they underlined the potential of PHASE 2 in the treatment of obesity and hypertriglyceridemia (Udani et al., 2004; Udani & Singh, 2007).

Different conclusions were reached by Celleno et al. (2007). Their results showed that the extract of *P. vulgaris* significantly contributes to reduction of body weight, BMI, fat mass, adipose tissue thickness, and waist, hip and thigh circumferences in overweight treated subjects. At the same time the lean body was preserved.

A similar study was carried out in 2010 on overweight men and women (Wu et al., 2010) in which a statistically significant difference was recorded in reduction of body weight and waist circumference in an active group compared with a placebo group. On the other hand, no significant change in hip circumference was recorded.

More recently PHASE 2 was tested for its efficacy in WL and weight maintenance (WM) in subjects with BMI between 25 and 35 kg/m^2^ (Grube et al., 2014).

This research is of particular importance for the sample size of the WL trial and for the duration of WM study (the largest sample, 123 subjects, and the longest single clinical trial, 24 weeks, respectively) conducted on *P. vulgaris* extract.

It proves the efficacy of the product in WL after 12 weeks (2.91 ± 2.63 kg vs. 0.92 ± 2.00 kg, *p* < .001) and in WM after 24 weeks (the mean weight of the subjects at week 24 was 99.34 ± 2.96% of baseline weight).

The growing interest in this research field is emphasized by a very recent paper that studies WL in obese subjects after the intake of 2,400 mg of *P. vulgaris* extract before each daily meal in a short period (35 days) (Wang et al., 2020). The differences in WL between the active and placebo group were significant (*p* < .1; 2.24 kg vs. 0.29 kg). BMI decreased by an average of 0.79 kg/m^2^ and the body fat decreased by 1.53% on average compared with the baseline. Other recorded parameters were thickness of subcutaneous fat, waist, and hip circumference that significantly decreased after the treatment.

These studies demonstrated the effectiveness of the commercial product PHASE 2 in the treatment of obesity. Weight loss, reduction in BMI, and decrease in waist circumference are the main effects of taking this food supplement.

However other important issues have been investigated to evaluate the effect of *P. vulgaris* extract in reducing postprandial plasma glucose, hyperglycemia, and insulin response.

### 3.6 Effect of α‐AI1 on hyperglycemia and hyperinsulinemia in humans

One of the obesity‐related diseases is diabetes mellitus, a metabolic disorder characterized by chronic hyperglycemia. Many therapies have been proposed by the scientific community to treat type 2 diabetes; the use of α‐AI is one of the effective therapeutic strategies to lower postprandial blood glucose levels (Agarwal & Gupta, 2016). In fact, as summarized in Table 3, the use of α‐AIs to reduce hyperglycemia and hyperinsulinemia had already been studied since 1973 on rats, dogs, and healthy humans (Puls & Keup, 1973).

**TABLE 3:** Main clinical trials on humans for the treatment of hyperglycemia and hyperinsulinemia with *Phaseolus vulgaris* extract

Reference	Administration	Subjects	Side effects	Main results
Layer, Rizza, et al. (1986)	Partially purified extract	Healty and diabetics	Abdominal discomfort and diarrhea	Decrease in postprandial plasma glucose and insulin concentration in both subjects
Boivin et al. (1987)	Partially purified extract	Healthy	Diarrhea	Reduction in starch digestion and postprandial glucose
Yamada, Yamamoto, and Yamaguchi (2007)	PHASE 2® + *Coleus forskohlii* extract + mushroom chitosan.	Diabetic	No side effects	Reduction in blood sugar levels and insulin levels
Udani, Singh, Barrett, and Preuss (2009)	PHASE 2	Healthy	No side effects	Reduction of GI after the administration of 2 and 3 g of PHASE 2®
Vinson, Al Kharrat, and Shuta (2009)	PHASE 2	Healthy	No side effects	A faster return to the plasma glucose base line after a meal
Zulkarnain, Setiawati, and Setiabudy (2009)	PHASE 2 + acarbose	Healthy	Not available	Reduction in postprandial glucose concentrations
Spadafranca et al. (2013)	Partially purified extract	Healthy	No side effects	Reduction in glucose, insulin, and C‐peptide levels. Reduce the desire to eat, prolonging the sense of satiety.

As mentioned above, both Boivin et al. (1987) and Layer, Rizza, et al. (1986) showed that a partially purified amylase inhibitor was able to decrease postprandial plasma glucose and insulin concentration in both healthy and diabetic subjects.

More recently, a new commercial supplement produced with the intent of preventing obesity and diabetes named “Super Bows Diet Type B” has been tested in Barrious Laboratories (Yamada et al., 2007). This granular supplement contains PHASE 2, *Coleus forskohlii* extract and mushroom chitosan.

In a double‐blind crossover test including 13 men and women with a fasting blood‐glucose level above 126 mg/L, the active product or the placebo (in a single dose) were administered 5 min before eating 300 g of polished rice. The analysis of blood glucose levels measured before and after eating (30–120 min later) showed that blood sugar levels and insulin levels were significantly lower in the active group as compared with the placebo group measured respectively 30 minu and 30–60 after meal intake. Unfortunately, these results have not been published in a peer‐reviewed journal.

On the other side, in 2009 three different independent papers focused on the same topic were published. In the first study differences in GI were investigated after the intake of white bread and butter with and without the addition of PHASE 2 (Udani et al., 2009). The GI is a scale that represents the rise of glucose level after food intake and is defined as “the incremental area under the blood glucose response curve of a 50 g carbohydrate portion of a test food expressed as a percent of the response to the same amount of carbohydrate from a standard food taken by the same subject”.

PHASE 2 was administered to 15 randomized volunteers with BMI between 18 and 25 (only 13 were considered in the final analysis) in a capsule or powder form at the dosage of 1,500, 2,000, and 3,000 mg. The GI was calculated using standard capillary glucose measurements.

The capsules with 2,000 and 3,000 mg of PHASE 2 reduced GI significantly, while the powder formulation was effective only with the highest dose administration.

The second study included two different placebo‐controlled, cross‐over tests (Vinson et al., 2009).

In the first trial, four slices of white bread and 42 g of margarine with or without 1,500 mg of PHASE 2 were administered to 11 normoglycemic subjects. With a clinical procedure, the blood was drawn before the meal and after every 15 min for 2 h. A faster return to the plasma glucose base line (62 vs. 80) was found in the active group compared with the placebo group and it was suggested that only 1/3 of the carbohydrates in the bread were absorbed with PHASE 2.

In the second trial, seven subjects consumed a frozen dinner of country fried steak with gravy, mashed potatoes, green beans, and cherry‐apple pie with or without a lower PHASE 2 dose (750 mg). Blood was sampled every 10 min for 60 min then periodically until 2 h.

Also in this case, the active group returned to the baseline faster than untreated subjects (58 vs. 70 min) and only 2/3 of the carbohydrates was absorbed using PHASE 2.

In the third study, the hypoglycemic power of bean extract was also investigated in combination with acarbose on 12 healthy volunteers. The subjects were administered 1,500 mg *P. vulgaris* extract and 50 mg acarbose, or acarbose 50 mg alone, before a carbohydrate meal. However, no significant differences were found between the two groups in reducing postprandial glucose level (Zulkarnain et al., 2009).

In a more recent trial, the efficacy of *P. vulgaris* extract (Beanblock; Indena S.p.A., Milan, Italy) was investigated both on glycometabolic and appetite control (Spadafranca et al., 2013). In this double‐blind, randomized, cross‐over study, 20 healthy fasting volunteers were asked to eat a standardized meal consisting of a sandwich of white bread, ham, oil, and tomato with 100 mg of active or placebo tablet. The blood was drawn at baseline and periodically for 3 h after meal to record glucose, insulin, C‐peptide, and ghrelin concentration.

The intake of the bean extract reduced the glucose response to the meal, accompanied by a lower insulin level and C‐peptide excursions. The sense of satiety was significantly reduced in the placebo group compared with the active one (third hour after meal) as a result of the suppression of ghrelin secretion inducing a lower desire to eat in the active group.

Several studies have proved the efficacy of *P. vulgaris* extract in reducing postprandial blood glucose concentration and so it is suggested as a valuable tool to prevent the onset of diabetes (Mahmood, 2016).

PHASE 2 is effective in reducing blood glucose and insulin levels in both healthy and diabetic subjects confirming what has been observed in preliminary animal studies and early human trials. As noted above, the side effects of this supplement are mostly attributable to transient or even absent episodes of diarrhea.

However, more studies are necessary to explore this topic in more depth in order to resolve some discrepancies among the findings. This research field is in any case still open and the consumption of beans has even been proposed as a possible low‐cost approach to reduce some ophthalmic disease such as cataract in patients with type 2 diabetes (Longo‐Mbenza & Muaka, 2013).

## 4 SAFETY

It should be noted that beans and their extracts also contain anti‐nutritional compounds. Trypsin inhibitors, lectins such as phytohemagglutinin, polyphenols (condensed tannins and anthocyanins), and some oligosaccharides, in fact, counteract the beneficial effects deriving from their consumption (Los, Zielinski, Wojeicchowski, Nogueira, & Demiate, 2018). The presence of these substances may interfere with digestibility and availability of nutrients (Worku & Sahu, 2017). Many studies have reported the occurrence of side effects and risk of adverse reactions after the ingestion of raw beans in humans and animals (Carmalt, Rosel, Burns, & Janzen, 2003; de Moya et al., 2003; Marzo, Alonso, Urdaneta, Arricibita, & Ibáñez, 2002).

Particular attention was given to PHA that, if administrated at high concentrations, causes hyperplastic growth of the small intestine, lower fractional rate of protein synthesis in skeletal muscle, and overgrowth of *Escherichia coli* in the lumen in a dose‐dependent manner (Bardocz et al., 1992; Pusztai et al., 1993).

With the aim of removing the activity of antinutritional compounds, different techniques have been reported and summarized by Kumar, Verma, Das, Jain, and Dwivedi (2013) in a recent review. For example, it has been observed that PHA is inactivated by cooking (Nciri et al., 2015; Rodhouse, Haugh, Roberts, & Gilbert, 1990; Thompson, Rea, & Jenkins, 1983). Unfortunately, this treatment also inactivates α‐AIs.

Furthermore, since hemagglutination activity greatly differs among all cultivars (Burbano, Muzquiz, Ayet, Cuadrado, & Pedrosa, 1999; Grant, More, McKenzie, Stewart, & Pusztai, 1983) and the α‐AI is more active on some than others (Bosi et al., 2019; Ghorbani et al., 2018; Wang et al., 2011; Yao, Hu, Zhu, Gao, & Ren, 2016), it is crucial to choose a cultivar with less PHA concentration and more α‐amylase activity as a raw material to produce bean extract.

However, the role of PHA in body WL is still debated. In fact, the intake of raw bean extract mixed in the diet with PHA lower than 0.2 g/kg leads to the reduction in body fat and body weight in obese Zucker rats (Pusztai et al., 1998). This group lost more fat compared with the control group without loss of skeletal muscle and body proteins.

A higher concentration (0.4 g/kg) of PHA was tolerated if accompanied by a higher lipid dose in the diet. Based on this evidence bean lectin has been suggested as a dietary adjunct to tackle obesity in a safe and effective dose range. However, when rats were fed with a high concentration of PHA and low in α‐AIs, there was no hypoglycemic effect (Bardocz, Grant, Pusztai, Franklin, & Carvalho, 1996).

Therefore, the attention has turned to the preparation of *P. vulgaris* extract with a dual action: (i) inhibition of α‐amylase and ii) anorexigenic effect caused by PHA (Fantini et al., 2009).

PHASE 2 is instead prepared using a special process to inactivate hemagglutination power and trypsin inhibiting activity and many studies tested its safety. The finished product contains less than 700 hemagglutinating activity units (HA) per gram.

Acute and subacute toxicity on rats was investigated by several studies (Chokshi, 2006, 2007; Harikumar et al., 2005). No significant side effects or mortality was recorded in these trials. The toxicity level was considered acute when it exceeded the highest dose tested in acute administration corresponding to 5 g PHASE 2/kg BW (Obiro et al., 2008).

Moreover, the absence of side effects related to the presence of PHA confirmed the product safety. The effects of subchronic (30 days treatment) and chronic (24 weeks) use of PHASE 2 administration were studied on humans too, and no side effects were recorded (Celleno et al., 2007; Grube et al., 2014).

Despite most studies are focused on PHASE 2, it is not the only α‐AI commercially available. In 2020 another carbo‐blocker extracted from *P. vulgaris* called Max Bloc® was tested (Zhang, Cavender, & Allen, 2020). Compared with three other commercial carbo‐blockers from China and the United States, it has a α‐amylase inhibitory activity 10–16 times higher and no hemagglutinin toxicity (0 HAU/g vs. 256,000–640,000 HAU/g of the others).

Moreover in vitro studies have shown that the ingestion of Max Bloc reduces starchy food digestion by 69.2% to 93.3%. Based on this evidence, this supplement has been proposed as a valid candidate in weight management and glucose control.

On the whole, α‐AI commercial products showed different characteristics probably linked to the different extraction procedures. These could also affect the real dose administered and the resulting effectiveness on the patient. The joint action of α‐AI and PHA may improve the effectiveness of the product in a dose‐dependent manner. Nevertheless, many of these studies look preliminary, thus more experiments should improve the safety and efficacy of these commercial products.

## 5 THE MODULATION OF GUT MICROBIOTA

The increased awareness of the role of gut microbiota in human and animal health is leading to more investments and research on this topic (Conlon & Bird, 2015). It has been demonstrated that the diet plays a preponderant role in the modulation of microbiota, even more important than genetic factors (David et al., 2014).

It has been proved that the gut microbiota composition changes in lean and obese individuals thus revealing that obesity is associated with the reduction in the abundance of *Bacteroidetes* and with the increase in the abundance of *Firmicutes* (Turnbaugh et al., 2006).

Based on this evidence, some recent studies have focused their attention on the effects that *P. vulgaris* extracts may have on microbiota composition in mice (Shi et al., 2020; Song et al., 2016).

Shi et al. (2020) investigated the role of α‐amylase extract in body weight, serum lipid levels, and gut microbiota composition in obese rats. During the experiment, three different doses of extract (0.5, 1, and 1.5%) were added to the diet and administered to induced obesity in rats for 10 weeks. At the end of experiments, rats fed with the two highest doses of extract showed a significantly lower BW. Under these conditions, abdominal fat accumulation, serum TC, and serum low density lipoprotein (LDL) levels were significantly reduced.

Interesting considerations have been made about the relationship among colonic content, pH, and the concentration of short‐chain fatty acid (SCFA). Administration of medium and high dose of bean extract significantly increased the colonic content compared with obese and non‐obese rats and led to a decrease in pH values of the colonic content (Shi et al., 2020).

This pH reduction is closely related to the increase of SCFA in intestinal content arising from the fermentation of carbohydrate by gut microbiota (Harris, Edwards, & Morrison, 2017; Poeikhampha & Bunchasak, 2010). The authors underlined the importance of SCFA in metabolic health, recalling that propionic acid (increased by medium and high doses) has an important role in the reduction of lipids level (Laparra & Sanz, 2010), thus suggesting that the lipid‐lowering effects resulting from the administration of *P. vulgaris* extract may be related to this increase in propionic acid.

Important changes were also recorded in gut microbiota after the administration of different diets. The high‐fat diet of the mice involved a reduction of diversity and richness in gut microbiota composition. However, the administration of *P. vulgaris* extract involved a reduction of the relative abundance of *Firmicutes* and *Proteobacteria*, and an increase of the relative abundance of *Bacteroidetes*, *Butyricoccus*, *Blautia*, and *Eubacterium* (phylum level). The latter groups of bacteria are SCFA‐producing, protecting the intestinal gut from damage preventing the onset of obesity and related diseases.

These conclusions are in agreement with what was found previously by Song et al. (2016) in the first study on the change in gut microbiota in diet‐induced obese mice by administration of kidney beans extract. Similarly, rats have been divided into three groups (fed with low‐fat diet, high‐fat diet, and high‐fat diet added with *P. vulgaris* extract) to monitor the body weight, serum lipid level, hepatic steatosis, and change in gut microbiota composition for 14 weeks.

Even in this case, the *P. vulgaris* extract treatment reduced body weight and food intake compared with animals fed with only high‐fat diet. The high‐fat diet involved an increase in TC (LDL, high density lipoprotein [HDL]), and serum triacylglycerols, while the administration of bean extract led to a reduction TG, TC, LDL but not HDL. The bean extract turned out to have an anti‐hepatosteatosis effect and to cause a decrease in glucose blood levels in rats fed with a high‐fat diet.

In accordance with the other study, the high‐fat diet induced a significance increase of *Firmicutes* and decrease in *Bacteroidetes*, *Proteobacteria*, and *Verrucomicrobia*, while bean extract involved a decrease in the relative abundance of *Firmicutes*, but a significant increase in *Verrucomicrobia* and *Actinobacteria*. The increase of the relative abundance of *Bifidobacterium*, *Lactobacillus*, and *Akkermansia* was also recorded at the phylogenetic genus level.

So, it is evident that the subjects receiving the extract benefit both in terms of body WL, reduced blood glucose level, and intestinal health.

In addition, Wang et al. (2021) investigated the modulation of gut microbiota in high‐fat fed C57BL/6J mice after 8 weeks of administration of *P. vulgaris* extract, yogurt, and a mixture of *P. vulgaris* extract and yogurt (YPVE) as additive feeding (Wang et al., 2021).

At phylum level, the Bacteroidetes and Proteobacteria in YPVE group was declined comparing with the other groups, while the abundance of *Firmicutes* in YPVE group remained higher than high‐fat fed mice. The YPVE mice microbiota was characterized by richness and abundance of microbial species, suggesting that the YPVE led to a healthy direction on the regulation of intestinal flora.

In summary, the intake of *P. vulgaris* extract in mice has the potential to modulate gut microbiota. The microbiota of treated animals was characterized by a greater richness in species. It was recorded an increase of the relative abundances of *Bacteroidetes* and *Akkermansia* and a reduction of *Firmicutes* and *Proteobacteria*, with the proliferation of SCFA‐producing bacteria, which reflects positively on the well‐being of the host. These findings are in fact giving further impetus to research in this field. Many recent works are focused on the role of *P. vulgaris* intake in microbiota modulation in animal models (Dias et al., 2019; Gomes Mariana et al., 2021; Kilua et al., 2020). Human model should be the next step for further research, in order to confirm the health effects of *P. vulgaris* extract also at the intestinal level.

## 6 CONCLUSIONS AND FUTURE PERSPECTIVE

Many natural products are potentially useful for human health and can be extracted in sufficient quantities from natural sources. Among these substances, we can include the protein inhibitors of the mammalian α‐amylase enzyme. Over the past 20–30 years, a large number of studies have been conducted that show the potential usefulness of these inhibitors, extracted from the seed of *P. vulgaris*, in producing strategies to contain the absorption of complex carbohydrates. The results of many clinical trials have generated encouraging results so as to induce the main American and European Control Authorities to issue authorizations to market common bean‐based preparations. Today, we can consider these preparations safe for human health although transient side effects can occur depending on the ingested doses. In this perspective, this review work could encourage further studies to better explore these issues, potentially affecting the real clinical efficacy of the commercial products (Izzo, Hoon‐Kim, Radhakrishnan, & Williamson, 2016).

A further future perspective is to use genetic approaches to isolate *P. vulgaris* lines devoid of active antinutritional lectin to product commercial snack, such as biscuits and crackers, maintaining α‐AI activity (Sparvoli et al., 2021).

Over 90% of the world's bean production is done in developing countries. A challenge that awaits us in the coming years is to select among the thousands of cultivars of *P. vulgaris* present in the world those with the most favorable inhibitor/anti‐nutritional factor ratio, and whose cultivation can respond to criteria of greater sustainability for the environment.

## CONFLICT OF INTEREST

The authors declare no conflicts of interest.

## Data Availability

Data sharing is not applicable to this article as no new data were created or analyzed in this study.
